# Entropy Generation and Consequences of Binary Chemical Reaction on MHD Darcy–Forchheimer Williamson Nanofluid Flow Over Non-Linearly Stretching Surface

**DOI:** 10.3390/e22010018

**Published:** 2019-12-22

**Authors:** Ghulam Rasool, Ting Zhang, Ali J. Chamkha, Anum Shafiq, Iskander Tlili, Gullnaz Shahzadi

**Affiliations:** 1 School of Mathematical Sciences, Zhejiang University, Yuquan Campus, Hangzhou 310027, China; grasool@zju.edu.cn (G.R.); zhangting79@zju.edu.cn (T.Z.); 2 College of Mathematics and Computer Science, Zhejiang Normal University, Jinhua 321004, China; 3 Mechanical Engineering Department, Prince Mohammad Bin Fahd University, Al-Khobar 31952, Saudi Arabia; achamkha@yahoo.com; 4 School of Mathematics and Statistics, Nanjing University of Information Science and Technology, Nanjing 210044, China; anumshafiq@ymail.com; 5 Department for Management of Science and Technology Development, Ton Duc Thang University, Ho Chi Minh City 758307, Vietnam; 6 Faculty of Applied Sciences, Ton Duc Thang University, Ho Chi Minh City 758307, Vietnam; 7 Department of Mechanical Engineering, École de Technologie Supérieure, ÉTS, Montreal, QC H3C 1K3, Canada; gullnaz.shahzadi.1@ens.etsmtl.ca

**Keywords:** Williamson nanofluid, magnetohydrodynamic, nonlinear stretching, porous medium, entropy generation

## Abstract

The current article aims to present a numerical analysis of MHD Williamson nanofluid flow maintained to flow through porous medium bounded by a non-linearly stretching flat surface. The second law of thermodynamics was applied to analyze the fluid flow, heat and mass transport as well as the aspects of entropy generation using Buongiorno model. Thermophoresis and Brownian diffusion is considered which appears due to the concentration and random motion of nanoparticles in base fluid, respectively. Uniform magnetic effect is induced but the assumption of tiny magnetic Reynolds number results in zero magnetic induction. The governing equations (PDEs) are transformed into ordinary differential equations (ODEs) using appropriately adjusted transformations. The numerical method is used for solving the so-formulated highly nonlinear problem. The graphical presentation of results highlights that the heat flux receives enhancement for augmented Brownian diffusion. The Bejan number is found to be increasing with a larger Weissenberg number. The tabulated results for skin-friction, Nusselt number and Sherwood number are given. A decent agreement is noted in the results when compared with previously published literature on Williamson nanofluids.

## 1. Introduction

Based on their properties, over the years, the fluids have been categorized into sub-categories. The most recent class of fluids is called nanofluids, which was introduced by Choi [1] in early 1995. Such fluids are a colloidal mixture of metallic nano-size particles and a base fluid. The metallic ingredients help in improvement of the thermo-physical properties of fluid under consideration. However, the nano-size of the particles allows us to consider the whole saturation as a fluid, called nanofluids, that complies with the definition of non-Newtonian fluids. Since then, numerous research articles were reported discussing different properties in different industrial, engineering, physical, and mathematical aspects. For example, Rasool and Zhang [2] reported a steady incompressible radiative flow of nanofluids over Riga surface. The Lorentz forces generated by the Riga plate are active contributors to the fluid flow, heat, and mass transport in the said formulation. The MHD stagnation point flow of nanofluids was discussed by Bai et al. [3], discussing the variation in heat and mass transport. The model was enriched by various parameters especially the radiation parameter that certainly effects the heat flux. Jusoh et al. [4] incorporated the bvp4c method to solve the final governing equations of the flow model comprising of a Maxwell nanofluid flow over a convectively heated surface. Dogonchi et al. [5] discussed MHD flow of Cu-Water nanofluid flowing through cavity using CVFEM. Rasool et al. [6,7] reported on some interesting findings in Marangoni convection of nanofluids involving simple as well as Riga plates with various other important physical parameters, respectively. Some recent and interesting articles can be found in [8,9,10,11,12,13,14,15,16,17,18,19,20,21,22,23,24,25,26] and cross references cited therein.

The fluid flow caused by a stretching surface has manifold applications in various industrial and engineering setups that involve nanofluids in their production procedures. For example, melt-spinning, glass fiber manufacturing, cooling process of metallic plates, manufacturing of rubber bands, and plastic sheets are the well known applications of nanofluids that involve stretching surfaces. Skiadis [27] attempted a hydromagnetic fluid flow through solid surface. Later, Crane [28] reported an MHD flow induced by a deforming/stretching surface in two dimensions. In recent developments, one can see many articles addressing the problems that are based on linear and nonlinear stretching rates. Rasool et al. [29] reported a Darcy relation in nanofluids flow over nonlinearly stretching sheet/surface resulting some interesting variations in heat and mass transport. In another article, Rasool and Zhang [30] reported the characteristics of Darcy relation and MHD (Magnetohydrodynamics) together with Cattaneo-Christov theory of heat and mass flux over nonlinearly stretching surface. The results were obtained through homotopy approach. A correlation was given at the end of the study to summarize the relative variations in heat and mass flux. Sandeep et al. [31] assessed the dusty nanofluid flow past a stretching sheet theoretically. The characteristics of heat source/sink and inclined MHD in nanofluid flow driven by a linear stretching surface was reported by Hayat et al. [32]. The results were reported via the homotopy approach. Interesting curves of the thermal layer can be seen as an outcome. Ziaei-Red et al. [33] showed the importance of permeable surface in a nanofluid flow caused by stretching. The outcomes were in good agreement with the previous literature.

Entropy generation in mathematical models that are related with industrial and engineering applications of nanofluids, is one of the trending aspect these days and over the years it has received an utmost consideration in the research community. Several fluid models are available in the literature to explain the phenomena of entropy generation in fluid flow systems. Though the primary source of entropy generation lies in the unreversibility in the presence of low Reynolds number but the existence of larger Reynolds is yet another undeniable fact and it has association with hydrodynamics in the context of entropy generation. Numerous research articles have been reported in recent years discussing the flow profiles as well as the entropy generation in the prescribed models. For example, Afridi and Qasim [34] proposed a model comprising of nanofluid with the addition of thermal radiation and viscous dissipation by a moving needle discussing the entropy generation. Lopez et al. [35] reported a radiative flow past a vertical porous micro-channel. The velocity slip and entropy generation were given due emphasis. The involved nanofluid was supposed to be a mixture of aluminum and water as base fluid. As a result, entropy generation showed certain increments for augmented Buyoncy and radiative heat but a declination is noticed towards volume fraction of nano-particles and suction/injection. Characteristics of EMHD and entropy generation in a curvy micro-channel was reported by Liu et al. [36]. The numerical results have shown that the strength of magnetic field is an important factor together with intensity of electric field, cross section ratio, curvature ratio, and viscous dissipation. The results show that local entropy generation is a decreasing function with an away movement from surface. However, higher viscous dissipation is an increasing factor for the said phenomena. In their study, Wang et al. [37] reported an entropy generation of a heater with different operating factors. The second law of thermodynamics was used to analyze the dynamics of the heater. Via transient process, the results indicate that the entropy generation decreases. An important study was reported by Sonia et al. [38] for analysis of entropy generation in a fluid flow that gains momentum in a natural way bounded by a semi-annular enclosure. Four types of nano-particles namely copper, silver, copper oxide and gold have been involved to saturate the given base fluid. The Maxwell–Garnetts model and Brinkman model have been used to calculate the effective conductivity (thermal) and viscosity of the nanofluid. Reportedly, the larger Rayleigh number results in enhancement of entropy generation. Sumaira et al. [39] reported the prospects of entropy generation in Williamson nanofluids incorporating a model involving two rotating disks. Entropy generation is calculated through second law of thermodynamics. In their study, Khan et al. [40] discussed the entropy generation in Williamson nanofluids through porous medium with linear stretching, and joule heating. Vatanmakan et al. [41] reported a volumetric heating and entropy generation in a flow modeled through turbine. SST k-w relation and Eulerian description were used to simulate the problem numerically. The results were in agreement with the experimental data. The drag force is found decreasing when volumetric heating is implemented. Recently, Zhang et al. [42] has reported a convection of paramagnetic fluid involving the concept of thermo-magnetic. The entropy generation was discussed in a porous enclosure. Results are achieved through the Boltzman numerical method. The larger Reynolds is taken into account. The non-gravitational condition results in enhancement of mass flux and reduction of Bejan number.

Pseudo-plastic materials have extensive demand in the industry for their special properties. The wide use of such materials ranges in photographic films, melts and solutions of polymers with larger molecular weights, suspensions, expulsion of sheets, etc. Various models were proposed in the literature to discuss such kind of fluid flow but the complex nature of rheological systems limits the sufficiency of the Navier stokes equations. Models, such as those by Carreau, Cross, Ellis, and the power law model were reported in the literature to discuss the characteristics of such Pseudo-plastic materials. However, models such as the Powell–Eyring model and the Williamson model are worthy to cover the deficiency in the original Navier Stokes equations. Williamson [43] reported an experimental study supplemented by a model, named after him as Williamson model, for the above mentioned complex Pseudo-plastic materials. Later on, numerous studies were reported following the model proposed by Williamson with some fruitful results. Blasius [44] discussed the properties of momentum boundary layer formulated in the fluid flow over a flat surface. The concepts of Blasius [44] and Sakiadis [27] were combined with Williamson fluid by Ramesh et al. [45] using convective boundary conditions. The results were obtained through Homotopy. Khan et al. [46] reported an interesting study on Williamson nanofluid flow past a cone. The special case was discussed with plate as well. The study revealed that temperature profile reduces for larger values of Prandtl but a reduction is noticed towards a stronger thermophoretic force. Hayat et al. [47] reported an MHD analysis of Williamson fluid over nonlinear variable surface. Nadeem et al. [48] reported the fluid flow, heat, and mass transport mechanism over a stretching surface where the subject fluid was taken as Williamson fluid. Salahuddin et al. [49] reported flow of Williamson fluid over stretching surface using the theory of Cattaneo–Christov for heat and mass transfer developments. Whereas Soret and Dufour effects on Williamson fluid flow was reported by Hayat et al. [50] using convective conditions. The results indicate that thermal as well as solute Biot numbers are increasing factors for temperature field.

Inspired by the above literature, we targeted the aspects of binary chemical reaction, Arrhenius activation energy and the entropy generation in magnetohydrodynamic Darcy flow of Williamson nanofluid. The Brownian diffusion due to random motion of nanoparticles and the Thermophoresis phenomena are present due to the saturation of metallic nanoparticles. The medium is maintained over an infinite nonlinearly stretching surface along the *x*-axis. Numerical simulation of the problem gives graphical results that are plotted accordingly. Various interesting aspects of fluid flow, heat, and mass transport mechanism, the wall drag intensity and the entropy generation were analyzed on the basis of numerical data. The results are compared with previous literature on Williamson nanofluids.

## 2. Problem Formulation

**Figure 1:**
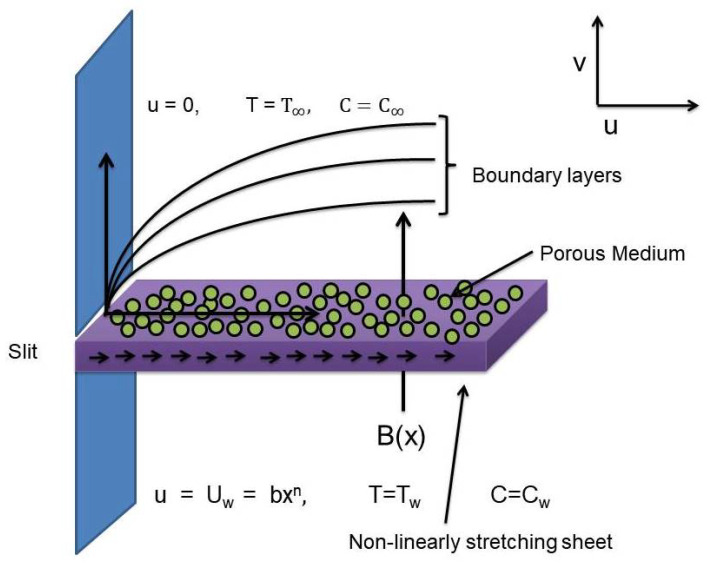
Physical diagram of the flow model.

Here we consider an incompressible, viscous and chemically reactive MHD Williamson nano-fluid flow maintained to flow through porous medium bounded by a non-linearly stretching surface in two dimensions (xy-coordinates). A binary chemical reaction with Arrhenius activation energy is considered. Uniform magnetic effect is induced but the assumption of tiny magnetic Reynolds number results in zero magnetic induction. The fluid flows alongside the *x*-axis given the velocity component *u* whereas no-displacement is taken alongside *y*-axis given the velocity component *v*. The sheet spreads nonlinearly along the positive *x*-axis with velocity $u=u_{w}=b\cdot x^{n}$ where n>1 represents the non-linearity in stretching and n=1 stands for the linear case. *b* is taken to be positive. Thermophoresis and Brownian diffusion is considered which appears due to the concentration of nanoparticles in base fluid as well as the random motion of the nanoparticles, respectively. A physical diagram of the flow model can be seen in Figure 1.

The modeled problem resembles the following governing equations (see for example Khan et al. [40]),

(1) $$\frac{\partial u}{\partial x}+\frac{\partial v}{\partial y}=0,$$

(2) $$\begin{array}{c} u\frac{\partial u}{\partial x}+v\frac{\partial u}{\partial y}=\nu \frac{\partial^{2}u}{\partial y^{2}}+\sqrt{2}\nu \Gamma \frac{\partial u}{\partial y}\frac{\partial^{2}u}{\partial y^{2}}-\left[\frac{C_{b}}{K^{1/2}}u-\frac{\nu }{K}\right]u-\frac{\sigma B_{0}^{2}}{\rho_{fl}}u, \end{array}$$

(3) $$u\frac{\partial T}{\partial x}+v\frac{\partial T}{\partial y}=\alpha_{fl}\frac{\partial^{2}T}{\partial y^{2}}+\tau \frac{D_{T}}{T_{\infty }}{\left(\frac{\partial T}{\partial y}\right)}^{2}+\tau D_{B}\left(\frac{\partial T}{\partial y}\frac{\partial C}{\partial y}\right)+\frac{\mu_{0}}{{\left(\rho c_{p}\right)}_{fl}}{\left(\frac{\partial u}{\partial y}\right)}^{2}+\mu_{0}\Gamma {\left(\frac{\partial u}{\partial y}\right)}^{3}+\frac{\sigma B_{0}^{2}}{{\left(\rho c_{p}\right)}_{fl}}u^{2},$$

(4) $$u\frac{\partial C}{\partial x}+v\frac{\partial C}{\partial y}=\frac{D_{T}}{T_{\infty }}\left(\frac{\partial^{2}T}{\partial y^{2}}\right)+D_{B}\left(\frac{\partial^{2}C}{\partial y^{2}}\right)-K_{r}^{2}{\left(\frac{T}{T_{\infty }}\right)}^{n_{1}}\exp\left(\frac{-E}{kT}\right)\left(C-C_{\infty }\right),$$

subject to following boundary conditions,

(5) $$u=u_{w}\left(x\right)=bx^{n},\;T=T_{w},\;C=C_{w},\;v=0,\;\mathrm{at}\;y=0,$$

(6) $$u\to 0,\;C\to C_{\infty },\;T\to T_{\infty }\;\mathrm{at}\;y\to \infty .$$

where $\nu \left(=\mu /\rho_{fl}\right)$ and μ are kinematic and dynamic viscosity of the base fluid, respectively, $\rho_{fl}$ is the given name of density of the base fluid, T,C stand for temperature field and concentration of nanoparticles, σ is designated symbol of electric conductivity, $\alpha_{fl}=k/{\left(\rho c\right)}_{fl}$ stands for thermal diffusivity, $D_{B}$ is given symbol for Brownian diffusion, τ is used as the given ratio of heat capacity of (fluid) ${\left(\rho c\right)}_{fl}$ to the heat capacity of (nanoparticles) ${\left(\rho c\right)}_{np}$, $T_{\infty },C_{\infty }$ are the typical ambient temperature field and concentration of nanoparticles, respectively. $D_{T}$ is given symbol of thermophoretic force. The uniformly induced magnetic field is given by $B_{0}$, $n_{1}$ is fitted strictly positive rate constant, *E* is used for activation energy and $K_{r}$ stands for binary chemical reaction. $\frac{C_{b}}{\sqrt{K}}=F$ is called the coefficient of inertia for the given porous medium. This term appears due to the drag force offered by the medium to fluid flow. Following Hayat et al. [11] define,

(7) $$\left.\begin{array}{c} v=-\sqrt{\frac{b\nu (n+1)}{2}}x^{\frac{n-1}{2}}\left(f\left(\eta \right)+\frac{n-1}{n+1}\eta f^{\prime }\left(\eta \right)\right),u=bx^{n}f^{\prime }\left(\eta \right),\\ \;\phi \left(\eta \right)=\frac{C-C_{\infty }}{C_{fl}-C_{\infty }},\theta \left(\eta \right)=\frac{T-T_{\infty }}{T_{fl}-T_{\infty }},\\ \;\eta =\sqrt{\frac{b(n+1)}{2\nu }}x^{\frac{n-1}{2}}y. \end{array}\right\}$$

By virtue of transformations, the final ODEs are given below,

(8) $$\begin{array}{cc} \; & f^{\prime \prime \prime }+ff^{\prime \prime }+\left(\frac{n+1}{2}\right)W_{1}f^{\prime \prime }f^{\prime \prime \prime }-\left(\frac{2n}{n+1}\right)f^{\prime^{2}}\\ \; & -\left(\frac{2n}{1+n}\right)\left(F_{r}\right){\left(f^{\prime }\right)}^{2}-\left(\frac{2}{n+1}\right)M_{1}^{2}f^{\prime }-\lambda \left(\frac{2}{n+1}\right)f^{\prime }, \end{array}$$

(9) $$\begin{array}{cc} \; & \theta^{\prime \prime }+\mathrm{Pr}\left(N_{b}\theta^{\prime }\phi^{\prime }+f\theta^{\prime }+{N_{t}\theta^{\prime }}^{2}+Ec\left(\frac{n+1}{2}\right){f^{\prime \prime }}^{2}+\frac{1}{\sqrt{2}}\left(\frac{n+1}{2}\right)EcW_{1}{f^{\prime \prime }}^{3}\right)+PrM_{1}^{2}Ec{f^{\prime }}^{2}=0, \end{array}$$

(10) $$\phi^{\prime \prime }+Sc\phi^{\prime }f+\frac{N_{t}}{N_{b}}\theta^{\prime \prime }-ScK_{1}N_{b}{\left(1+\delta_{1}\theta \right)}^{n_{1}}\exp\left[\frac{-E_{1}}{1+\delta_{1}\theta }\right]\phi =0,$$

such that,

(11) $$f=0,\;f^{\prime }=1,\;\theta =1,\;\phi =1,\;\mathrm{at}\;\eta =0,$$

(12) $$f^{\prime }\to 0,\;\phi \to 0,\;\theta \to 0\;\mathrm{as}\;\eta \to \infty .$$

Here $W_{1}$ is Weissenberg number, Pr is known for Prandtl, $M_{1}$ is known for magnetic field, $F_{r}$ is used for inertial force, λ is treated as porosity factor, $N_{t}$ is Thermophoresis whereas $N_{b}$ is Brownian diffusion, Sc is Schmidt factor, $K_{R}$ is chemical reaction, $E_{1}$ is the activation energy. Ec is used as a symbol for Eckert number. Prime ${"}_{{}^{\prime }}"$ denotes differentiation regarding η. The dimensionless expressions are,

(13) $$\left.\begin{array}{c} W_{1}=\frac{\sqrt{2}\Gamma b^{3/2}x^{\frac{\left(3n-1\right)}{2}}}{\sqrt{\nu }},\;\mathrm{Pr}=\frac{\nu }{\alpha },\;{\left(M_{1}\right)}^{2}=\frac{\sigma B_{0}^{2}}{b\rho_{fl}x^{n-1}},\;F_{r}=\frac{C_{b}x}{K^{1/2}},\;\lambda =\frac{2\nu }{Kbx^{n-1}},\\ N_{t}=\frac{{\left(\rho c\right)}_{p}D_{Th}\left(T_{fl}-T_{\infty }\right)}{{\left(\rho c\right)}_{fl}\nu T_{\infty }},\;N_{b}=\frac{{\left(\rho c\right)}_{p}D_{Br}\left(C_{fl}-C_{\infty }\right)}{{\left(\rho c\right)}_{fl}\nu },\\ Sc=\frac{\nu }{D_{Br}},\;K_{R}=K_{r}^{2}\left(C_{fl}-C_{\infty }\right),\;E_{1}=\frac{E}{kT_{\infty }},Ec=\frac{b^{2}x^{2n}}{c_{p}\left(T_{w}-T_{\infty }\right)}. \end{array}\right\}$$

## 3. Mathematical Modeling for Entropy Generation

The governing Entropy equation for the modeled problem can be formulated as followed, (see for example Wang et al. [37], Qayyum et al. [39] and Khan et al. [40]),

(14) $$\begin{array}{cc} E_{G}= & \frac{k}{T_{\infty }^{2}}{\left(\frac{\partial T}{\partial y}\right)}^{2}+\frac{\sigma }{T_{\infty }}B_{0}^{2}u^{2}+\frac{R_{D_{B}}}{T_{\infty }}\left(\frac{\partial T}{\partial y}\right)\left(\frac{\partial C}{\partial y}\right)\\ \; & +\frac{R_{D_{B}}}{C_{\infty }}{\left(\frac{\partial C}{\partial y}\right)}^{2}+\frac{\mu_{0}}{T_{\infty }}\left({\left(\frac{\partial u}{\partial y}\right)}^{2}+\Gamma {\left(\frac{\partial u}{\partial y}\right)}^{3}\right)+\frac{\mu_{0}}{T_{\infty }}\left(\frac{1}{\rho_{fl}K}u^{2}\right). \end{array}$$

Equation (7) in Equation (14) yields the following non-dimensional form:

(15) $$\begin{array}{cc} N_{G} & =\beta_{1}\left(\frac{n+1}{2}\right){\theta^{\prime }}^{2}+Br_{1}\left(\frac{n+1}{2}\right){f^{\prime \prime }}^{2}+Br_{1}\left(\frac{n+1}{2}\right)\frac{W_{1}}{\sqrt{2}}{f^{\prime \prime }}^{3}+M_{1}^{2}\left(\frac{n+1}{2}\right)Br_{1}{f^{\prime }}^{2}\\ \; & +L_{1}\left(\frac{n+1}{2}\right)\frac{\beta_{2}}{\beta_{1}}{\phi^{\prime }}^{2}+L_{1}\left(\frac{n+1}{2}\right)\theta^{\prime }\phi^{\prime }+Br_{1}\lambda \left(\frac{n+1}{2}\right){f^{\prime }}^{2}, \end{array}$$

where

(16) $$\begin{array}{cc} N_{G} & =\frac{E_{G}T_{\infty }\nu }{x^{n-1}k\left(T_{w}-T_{\infty }\right)b},\;\;\beta_{1}=\frac{T_{w}-T_{\infty }}{T_{\infty }},\\ \; & \beta_{2}=\frac{C_{w}-C_{\infty }}{C_{\infty }},\;\;Br_{1}=\frac{\mu_{0}b^{2}x^{2n}}{k\left(T_{w}-T_{\infty }\right)},\\ \; & L_{1}=\frac{R_{D_{B}}}{k}\left(C_{w}-C_{\infty }\right), \end{array}$$

are the entropy generation rate, temperature difference parameter, concentration difference parameter, Brinkman number, diffusion parameter, respectively.

## 4. Expressions for Physical Quantities

Wall drag and relevant flux numbers (Nusselt, Sherwood) are defined as follows,

(17) $$\left.\begin{array}{c} {Re}_{x}^{1/2}C_{fx}=\left(\frac{\sqrt{n+1}}{\sqrt{2}}\right)\left(f^{\prime \prime }\left(0\right)-W_{1}f^{{\prime \prime }^{3}}\left(0\right)\right),\\ {Re}_{x}^{-1/2}Nu_{x}=-\frac{\sqrt{n+1}}{\sqrt{2}}\theta^{\prime }\left(0\right),\\ {Re}_{x}^{-1/2}Sh_{x}=-\frac{\sqrt{n+1}}{\sqrt{2}}\phi^{\prime }\left(0\right), \end{array}\right\}$$

where ${Re}_{x}=bx^{n+1}/\nu$ is local Reynolds.

## 5. Numerical Solution of the Problem

The nonlinear problems (8–12) and (15) subject to the given boundary conditions formulate two-point BVPs. The system is solved through numerical shooting technique, transforming the BVPs into initial value problems (IVPs) first. The following procedure is adopted in this transformation.

### 5.1. The Governing Equations

The governing equations are, therefore, written as follows,

(18) $$\begin{array}{cc} \; & f=f,\\ \; & \frac{\partial f}{\partial \eta }=f^{\prime }=g,\\ \; & \frac{\partial^{2}f}{\partial \eta^{2}}=f^{\prime \prime }=g^{\prime }=h,\\ \; & \frac{\partial^{3}f}{\partial \eta^{3}}=f^{\prime \prime \prime }=g^{\prime \prime }=h^{\prime }=\left(\frac{2n}{n+1}\right)g^{2}-\left(\frac{n+1}{2}\right)W_{1}hh^{\prime }+\left(\frac{2n}{n+1}\right)F_{r}g^{2}+\left(\frac{2}{n+1}\right)M_{1}^{2}g+\lambda \left(\frac{2}{n+1}\right)g,\\ \; & \theta =\theta ,\\ \; & \frac{\partial \theta }{\partial \eta }=\theta^{\prime }=s,\\ \; & \phi =\phi ,\\ \; & \frac{\partial \phi }{\partial \eta }=\phi^{\prime }=t,\\ \; & \frac{\partial^{2}\theta }{\partial \eta^{2}}=\theta^{\prime \prime }=s^{\prime }=-Pr\left[N_{b}st+fs+N_{t}s^{2}\right]-Ec\left(\frac{n+1}{2}\right)Prh^{2}-\frac{1}{\sqrt{2}}\left(\frac{n+1}{2}\right)EcPrW_{1}h^{3}-M_{1}^{2}EcPrg^{2},\\ \; & \frac{\partial^{2}\phi }{\partial \eta^{2}}=\phi^{\prime \prime }=t^{\prime }=ScK_{R}N_{b}{\left(1+\sigma_{1}\theta \right)}^{n}\exp\left(\frac{-E}{1+\theta \sigma_{1}}\right)\phi -Sctf-\frac{N_{t}}{N_{b}}s^{\prime }. \end{array}$$

Such that,

(19) $$\begin{array}{c} f=0,\;g=1,\;\phi =\theta =1,\;\eta \to 0,\\ g\to 0,\;\phi \to 0,\;\theta \to 0,\;\eta \to \infty . \end{array}$$

### 5.2. Entropy Generation

Following the same procedure as above, the entropy equation is given as follows,

(20) $$\begin{array}{cc} N_{G} & =\beta_{1}\left(\frac{n+1}{2}\right)s^{2}+Br_{1}\left(\frac{n+1}{2}\right)h^{2}+Br_{1}\left(\frac{n+1}{2}\right)\frac{W_{1}}{\sqrt{2}}h^{3}+M_{1}^{2}\left(\frac{n+1}{2}\right)Br_{1}g^{2}\\ \; & +L_{1}\left(\frac{n+1}{2}\right)\frac{\beta_{2}}{\beta_{1}}t^{2}+L_{1}\left(\frac{n+1}{2}\right)st+Br_{1}\lambda \left(\frac{n+1}{2}\right)g^{2}, \end{array}$$

### 5.3. Physical Quantities

The physical quantities, i.e., Skin-friction, Nusselt and Sherwood numbers used in the numerical scheme are given below,

(21) $$\left.\begin{array}{c} {Re}_{x}^{1/2}C_{fx}=\left(\frac{\sqrt{n+1}}{\sqrt{2}}\right)\left(h^{2}-W_{1}h^{3}\left(0\right)\right),\;\eta \to 0,\\ {Re}_{x}^{-1/2}Nu_{x}=-\frac{\sqrt{n+1}}{\sqrt{2}}s,\;\eta \to 0,\\ {Re}_{x}^{-1/2}Sh_{x}=-\frac{\sqrt{n+1}}{\sqrt{2}}t,\;\eta \to 0. \end{array}\right\}$$

A careful selection of initial guesses is made to repeatedly solve the given IVPs using fourth order RK-method. Secant method is implemented to adjust the values of aforementioned three quantities for better approximation. A convergence criteria based on the difference of previous to current iteration is employed. For a difference equal or less than $10^{-5}$, the solution is treated convergent, thus the iterations are terminated thereafter.

## 6. Results and Discussion

### 6.1. Wall Drag Force, Heat Flux Rate and Mass Flux Rate

Velocity field, temperature distribution, concentration of nanoparticles, and entropy generation rate are described physically here in this section. A numerical scheme is applied to achieve the final solutions that are sufficient to depict the behavior of flow profiles. In Table 1, we organized the data obtained upon fluctuation of the values of various parameters such as porosity factor, magnetic parameter and Weissenberg number. We see an enhancement in the drag force for augmented values of Magnetic parameter whereas a decrement can be seen for Weissenberg number. In Table 2, we organized the data collected upon numerical simulation of the problem for local Nusselt and local Sherwood numbers. One can see that Brownian diffusion results in decay of heat transfer rate whereas, the variation in Prandtl number results in enhancement of the heat transfer rate. The mass flux rate enhances for augmented values of chemical reaction but decays for the Arrhenius activation energy parameter. The full data tables are very handy in industrial applications of nanofluids. The involvement of Darcy medium significantly reduces the fluid movement. It affects the heat and mass transport mechanism due to inertial force and porosity factor. The drag force increases due to the resistive nature of medium (porous medium). Table 3 gives a comparison on skin-friction data with previously published data. Table 4 is organized on the comparison of Nusselt data with Khan et al. [40].

### 6.2. Stream Functions

**Figure 2:**
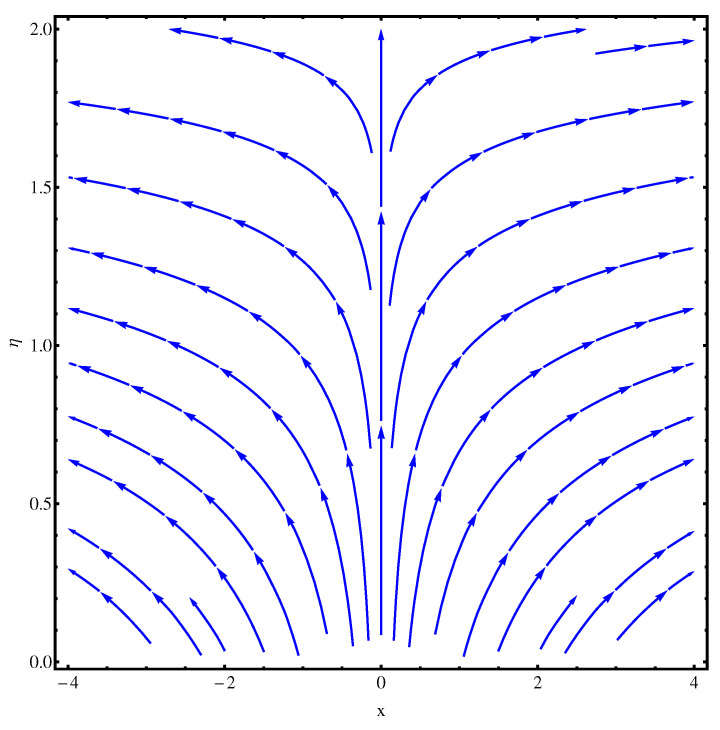
Stream functions at n=1.

**Figure 3:**
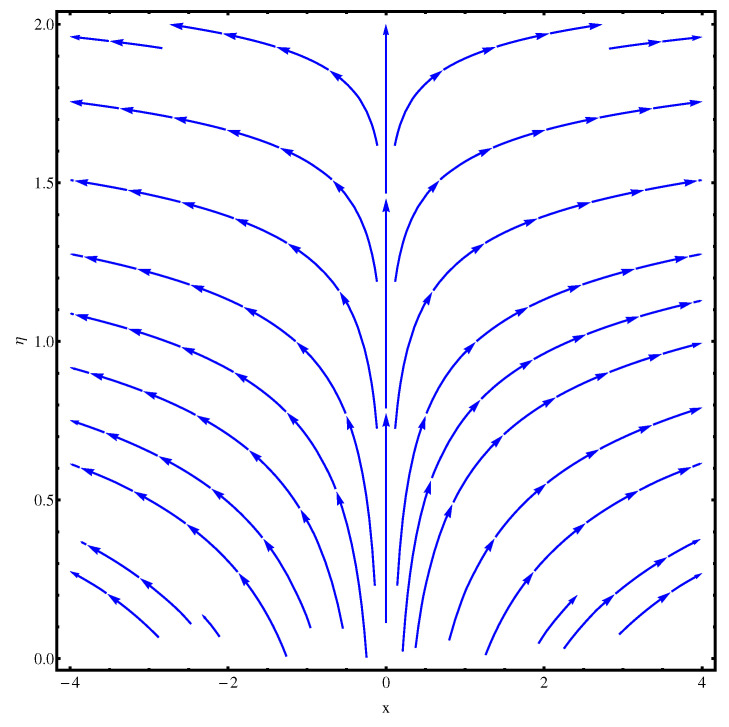
Stream functions at n=2.

The stream functions for the given flow model are plotted in Figure 2 and Figure 3. In Figure 2, the linear case of stretching is considered whereas, in Figure 3, the stretching is assumed to be nonlinear. In both the figures, a slight variation is noted in the stream curves. Smooth curves are noticed for linear case whereas, in nonlinear case the curves spread more from the origin compared to far away from the origin.

### 6.3. Velocity Field

**Figure 4:**
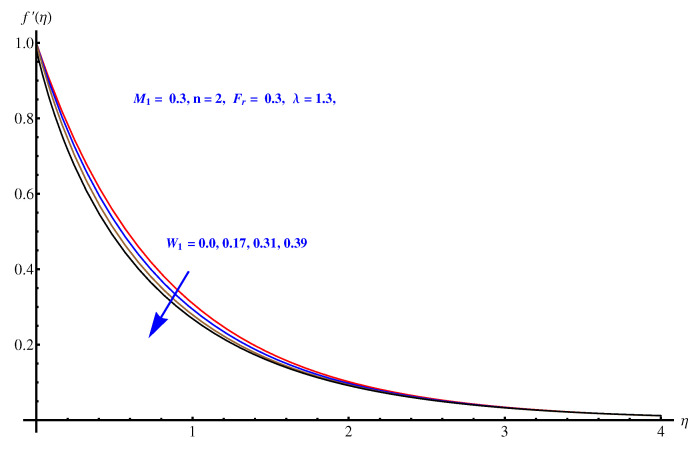
Influence of $W_{1}$ on velocity field.

**Figure 5:**
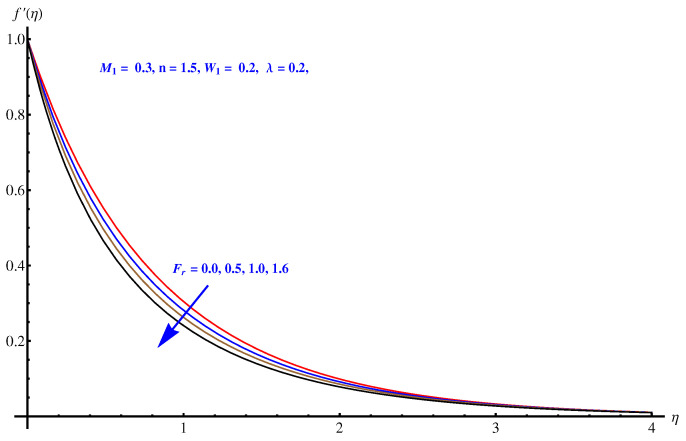
Influence of $F_{r}$ on velocity field.

**Figure 6:**
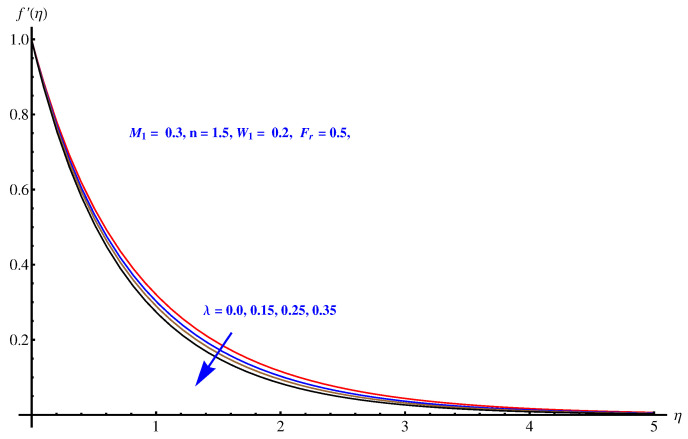
Influence of λ on velocity field.

We analyzed the velocity field for three parameters i.e., Weissenberg number, Inertial force parameter and porosity factor. One can see that the velocity field is decaying in the whole domain i.e., [0.0, 0.40] for the above mentioned three parameters. The inverse relation of these parameters with momentum equation is justified. For Weissenberg number we justify our graphical explanation given in Figure 4 with an argument that Weissenberg number is related with analyzing the viscoelastic flows where the role of relaxation time parameters is effective. Greater values of Weissenberg numbers are linked with more relaxation time which creates more resistance period for fluid motion. For inertial force parameter (see Figure 5), the sudden bumps in the way of fluid flow are the reasons for reducing the fluid motion. This is due to the resistive force active in the direction normal to the fluid flow. The porosity factor itself justifies the reduction in fluid motion (see Figure 6) due to its relation with frictional force and intensive drag force. The greater the porosity factor, the greater the friction and less motion of the fluid is expected.

### 6.4. Temperature Distribution

**Figure 7:**
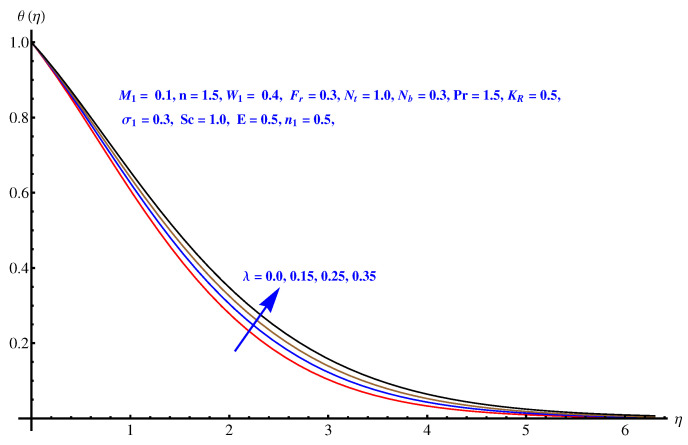
Influence of λ on temperature field.

**Figure 8:**
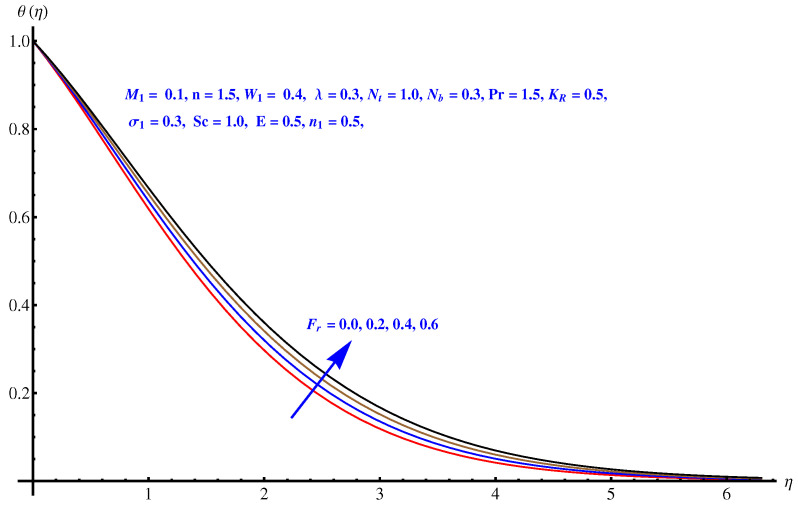
Influence of $F_{r}$ on temperature field.

**Figure 9:**
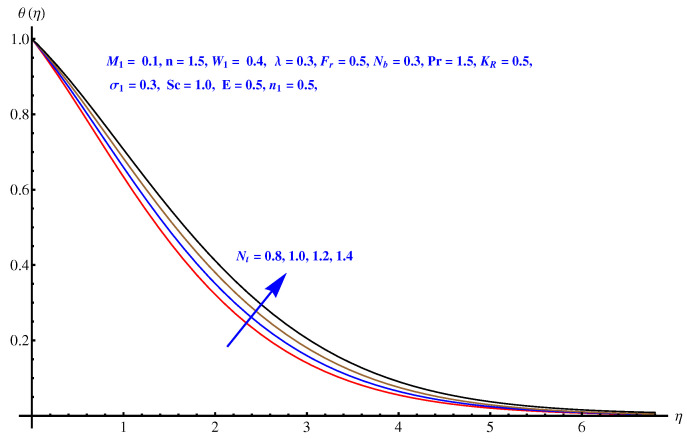
Influence of $N_{t}$ on temperature field.

**Figure 10:**
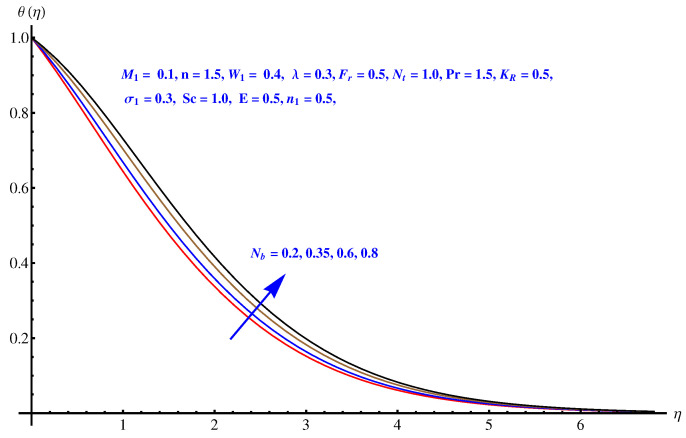
Influence of $N_{b}$ on temperature field.

From Figure 7, Figure 8, Figure 9 and Figure 10, the results are related to variation in temperature distribution for augmented values of porosity factor, Inertial force parameter, Thermophoresis, and Brownian diffusion. In particular, Figure 7 gives the graphical description of the influence of augmented values of λ on temperature distribution. The more intensive porosity factor results in more effective resistive force active on the way of fluid motion. This resistive force is the reason behind incremental trend in thermal profile and enhancement of the thickness of associated boundary layer. The variation in thermal profile due to Forchheimer number (inertial force parameter) is given in Figure 8. Clearly the more stronger inertial force results in more thermal distribution and the associated boundary layer thickness receives increment. Physically, the stronger inertial force is due to the intensive drag force coefficient “$C_{b}$”. For higher values of $C_{b}$ i.e., $\left(\frac{C_{b}}{\sqrt{K}}=F\right)$, stronger inertial force is effective within the model that enhances the collisions of fluid packets and rises the temperature field. Figure 9 and Figure 10 are the display of variation noted in thermal distribution due to the Thermophoresis and Brownian diffusion. Both the parameters are enhancing factors for the thermal layer. The reason behind this enhancement in temperature profile is the in-predictive motion of nanoparticles due to Brownian diffusion. The more intensive the thermophoretic force, the more abrupt the diffusion of the particles and this causes an increase in the thermal profile and associated boundary layer thickness receives increment.

### 6.5. Concentration Distribution

**Figure 11:**
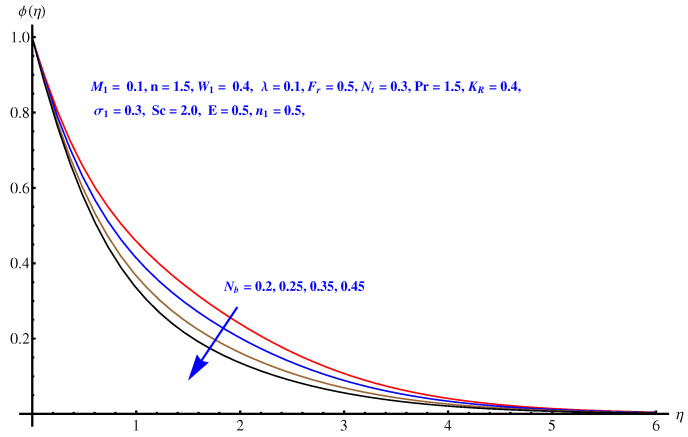
Influence of $N_{b}$ on concentration field.

**Figure 12:**
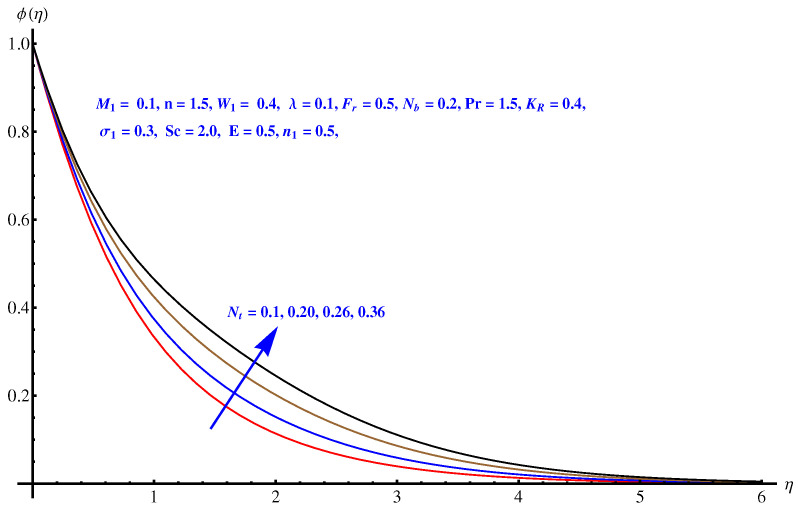
Influence of $N_{t}$ on concentration field.

**Figure 13:**
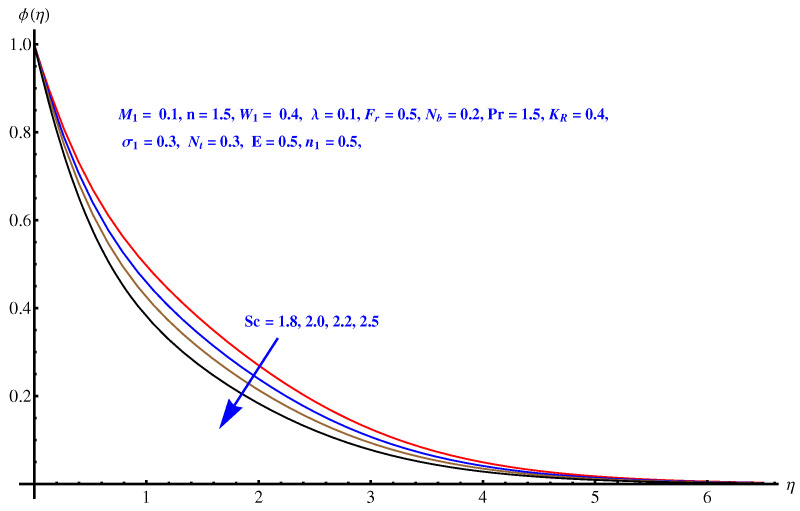
Influence of Sc on concentration field.

**Figure 14:**
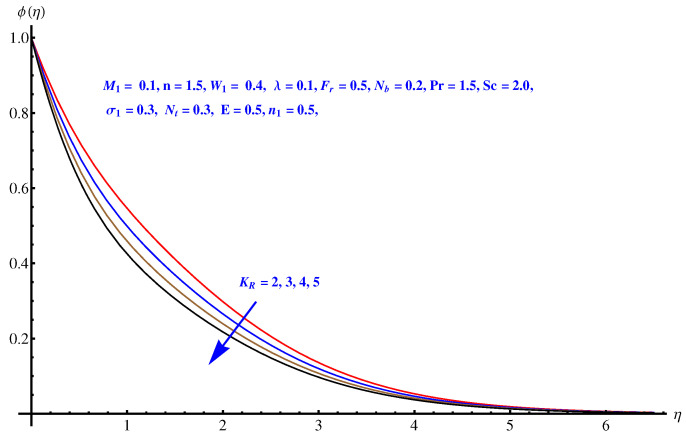
Influence of $K_{R}$ on concentration field.

**Figure 15:**
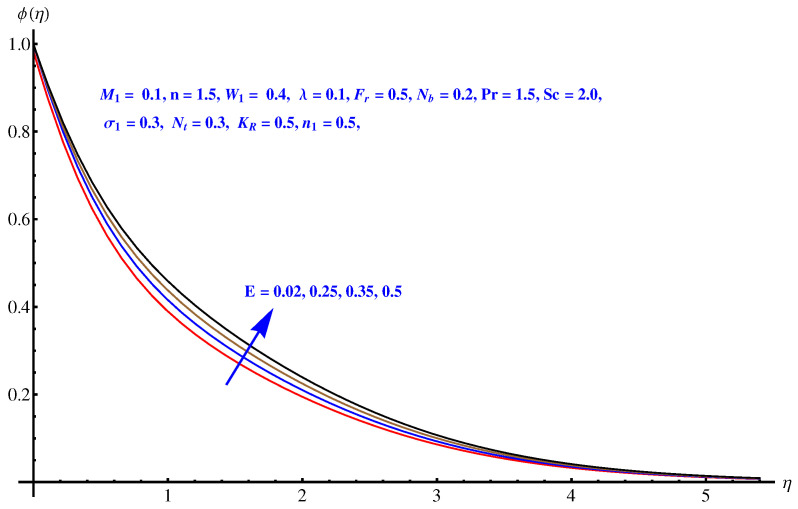
Influence of *E* on concentration field.

**Figure 16:**
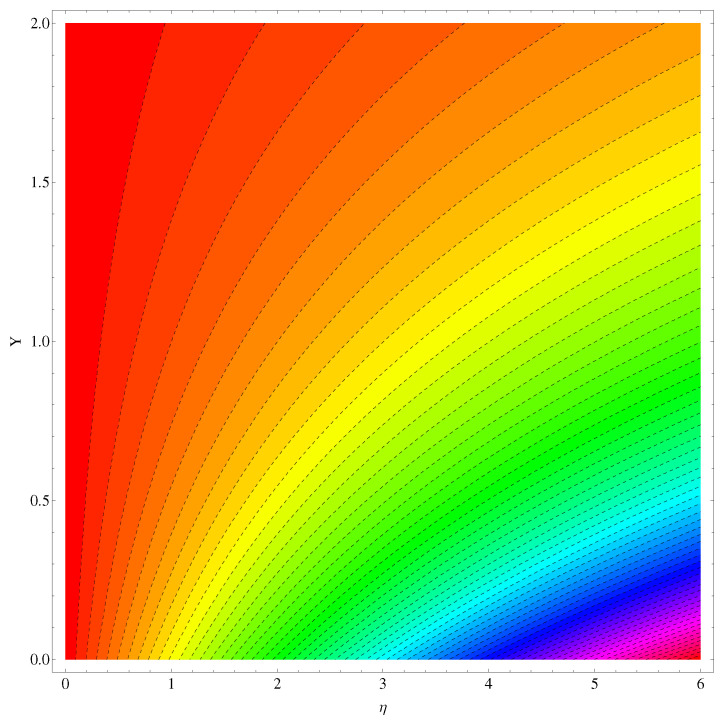
IG at n=1.

**Figure 17:**
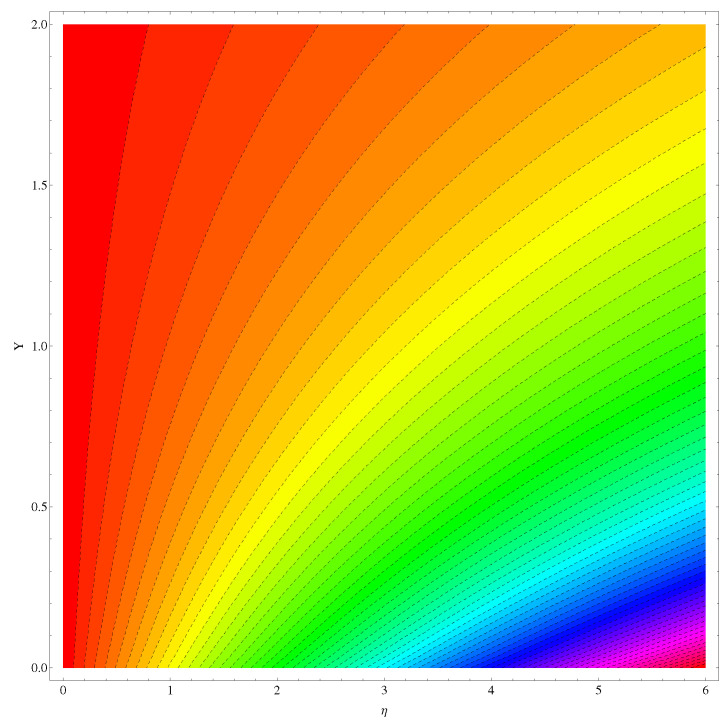
IG at n=2.

Here in this subsection we discuss the variations noted in concentration of the nanoparticles for various values of Brownian diffusion parameter, Thermophoresis parameter, Schmidt number, Chemical reaction, and Arrhenius activation energy parameter. The results are plotted graphically in Figure 11, Figure 12, Figure 13, Figure 14 and Figure 15. In particular, Figure 11 is the display of the impact of Brownian diffusion on concentration of the nanoparticles. In the whole domain i.e., [0.0, 0.50], the concentration of nanoparticles shows reduction for higher values of $N_{b}$ whereas, opposite behavior is noticed in concentration of nanoparticles with higher values of $N_{t}$. In the domain fixed for Schmidt number i.e., [0.0, 3.0], the concentration field increases. Mathematically, Schmidt factor is treated as a non-dimensional number relating mass diffusivity with momentum diffusivity yielding a fluid flow display. These two terms are physically called the mass transport layer and hydrodynamic thickness layer. For an enhancement in the Schmidt factor, the mass diffusion drops down which results in decrement in concentration field. The influence of chemical reaction and Arrhenius activation energy parameters is given in Figure 14 and Figure 15. For stronger chemical reaction, a destructive outcome is noted which decomposes the reactant species. Thus, the associated boundary layer reduces for augmented chemical reaction. An opposite trend is noted in case of Activation energy. Figure 16 and Figure 17 are the contour graphs at n=1 and n=2, respectively. Near the origin, the variation is less as compared to the free surface.

### 6.6. Entropy Generation

**Figure 18:**
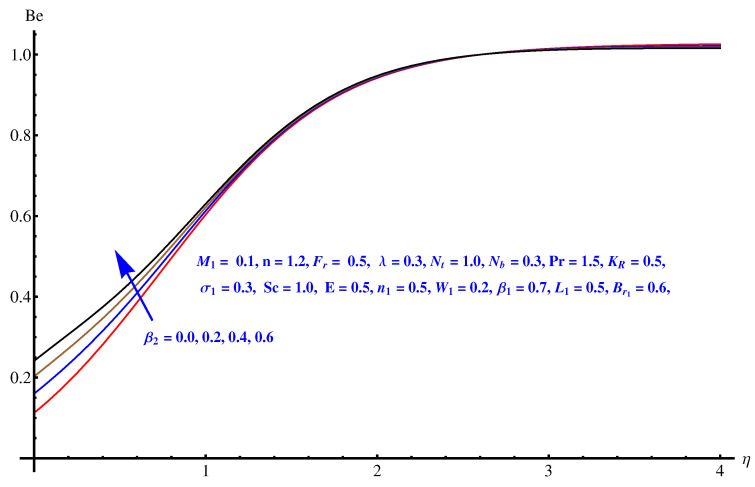
Be versus $\beta_{2}$.

**Figure 19:**
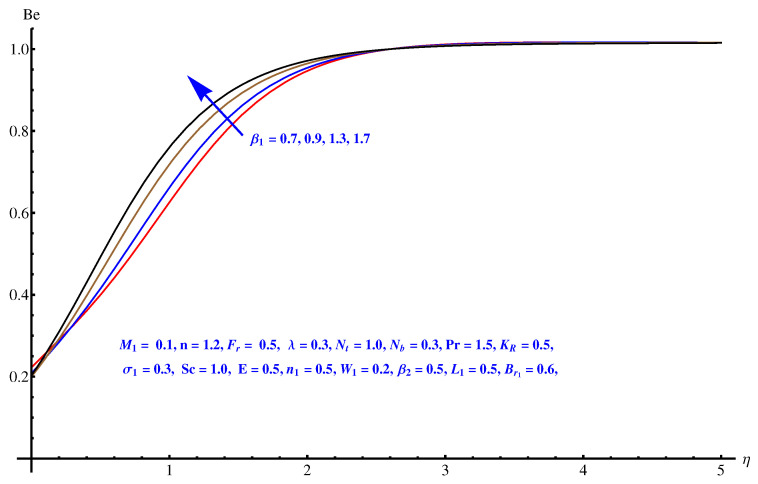
Be versus $\beta_{1}$.

**Figure 20:**
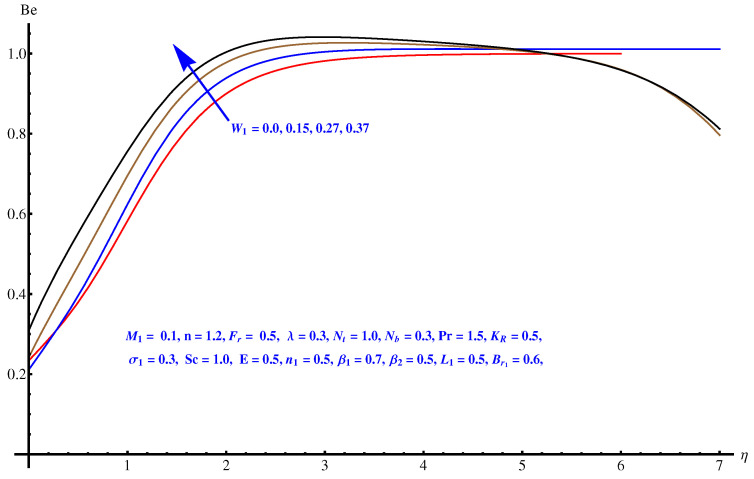
Be versus $W_{1}$.

The graphs plotted in Figure 18, Figure 19 and Figure 20 reflect the variations noted in Be for various values of $\beta_{1}$, $\beta_{2}$ and $W_{1}$ one by one. For augmented values of both the temperature difference parameter $\beta_{1}$ and $\beta_{2}$ we observe an enhancement in the respective magnitude of Be as displayed in Figure 18 and Figure 19. Figure 20 gives an explanation for the variation in Be with respect to the Weissenberg number ($W_{1}$). A clear enhancement can be seen in the interval [0.0, 0.40] for the Weissenberg number.

## 7. Conclusions

We considered an incompressible, viscous MHD Williamson nanofluid flow maintained to flow through porous medium bounded by a non-linearly stretching surface in two dimensions (xy-coordinates). A binary chemical reaction with Arrhenius activation energy is considered. Uniform magnetic effect is induced but the assumption of tiny magnetic Reynolds number results in zero magnetic induction. The governing equations are transformed into Ordinary equations. The nonlinear problems (8–12) and (15) subject to the given boundary conditions formulate two-point BVPs. The system is solved through numerical shooting technique, transforming the BVPs into initial value problems (IVPs) first. Herein, we calculated the entropy generation rate with a comprehensive analysis of the heat and mass transport mechanism, the wall drag intensity and the variation in flow profiles for various fluid parameters. Following the model given by Buongiorno, the modeling is done for a physical situation assumed under certain conditions. The salient features of this study are listed below:

- An enhancement in the drag force for augmented values of Magnetic parameter is noticed whereas a decrement can be seen for the Weissenberg number. The resistive Lorentz force active normal to the fluid flow is responsible for this behavior.
- The Brownian diffusion results in decay of heat transfer rate whereas, the variation in Prandtl number results in enhancement of the heat transfer rate.
- The mass flux rate enhances for augmented values of chemical reaction but decays for the Arrhenius activation energy parameter.
- For the stream functions, we see that smooth curves are produced for linear case whereas, in nonlinear case the curves spread more from the origin.
- Greater values of Weissenberg numbers are linked with more relaxation time which creates more resistance period for fluid motion.
- The more stronger inertial force results in more thermal distribution and the associated boundary layer thickness receives increment.
- In the whole domain i.e., [0.0, 0.50], the concentration of nanoparticles shows reduction for higher values of $N_{b}$ whereas, opposite behavior is noticed in concentration of nanoparticles with higher values of $N_{t}$.
- Schmidt factor is treated as a non-dimensional number relating mass diffusivity with momentum diffusivity yielding a fluid flow display. These two terms are physically called the mass transport layer and hydrodynamic thickness layer. For an enhancement in the Schmidt number, mass diffusion drops down which results in a decrement in the concentration field.

## Figures and Tables

**Table 1:** Skin friction at n=2 and $n_{1}=0.5$.

$W_{1}$	$M_{1}$	$F_{r}$	λ	$-C_{f}$
0.0	0.2	0.5	0.5	3.73592
0.2				3.06156
0.4				2.48776
0.2	0.0	0.5	0.5	3.02498
	0.2			3.06156
	0.4			3.31165
0.2	0.2	0.0	0.5	2.4912
		0.5		3.06156
		1.0		3.39709
0.2	0.2	0.5	0.0	2.61406
			0.5	3.06156
			1.0	4.13067

**Table 2:** Nusselt number (heat flux rate) and Sherwood number (mass flux rate) at $n=2,n_{1}=0.5$.

$W_{1}$	$M_{1}$	$F_{r}$	λ	Pr	$N_{t}$	$N_{b}$	Sc	$K_{R}$	$\sigma_{1}$	*E*	Nusselt	Sherwood
0.0	0.2	0.5	0.5	1.0	1.0	0.3	1.0	0.5	0.3	0.5	0.36517	0.726346
0.2											0.348383	0.742882
0.4											0.260131	0.829416
0.2	0.0	0.5	0.5	1.0	1.0	0.3	1.0	0.5	0.3	0.5	0.350179	0.741453
	0.2										0.348383	0.742882
	0.4										0.305662	0.788401
0.2	0.2	0.0	0.5	1.0	1.0	0.3	1.0	0.5	0.3	0.5	0.36404	0.727939
		0.5									0.348383	0.742882
		1.0									0.326917	0.765154
0.2	0.2	0.5	0.0	1.0	1.0	0.3	1.0	0.5	0.3	0.5	0.372632	0.72538
			0.5								0.348383	0.742882
			1.0								0.125845	0.867449
0.2	0.2	0.5	0.5	0.5	1.0	0.3	1.0	0.5	0.3	0.5	0.265106	0.835514
				1.0							0.348383	0.742882
				1.5							0.38281	0.728116
0.2	0.2	0.5	0.5	1.0	0.5	0.3	1.0	0.5	0.3	0.5	0.42002	0.719649
					1.0						0.348383	0.742882
					1.5						0.291001	0.793598
0.2	0.2	0.5	0.5	1.0	1.0	0.1	1.0	0.5	0.3	0.5	0.360205	0.0817931
						0.3					0.348383	0.742882
						0.6					0.285437	0.950296
0.2	0.2	0.5	0.5	1.0	1.0	0.3	0.5	0.5	0.3	0.5	0.376174	0.214407
							1.0				0.348383	0.742882
							1.5				0.335178	1.1016
0.2	0.2	0.5	0.5	1.0	1.0	0.3	1.0	0.1	0.3	0.5	0.379041	0.0418443
								0.3			0.359585	0.463542
								0.5			0.348383	0.742882
0.2	0.2	0.5	0.5	1.0	1.0	0.3	1.0	0.5	0.1	0.5	0.349899	0.684649
									0.4		0.347684	0.769771
									0.8		0.3452	0.865346
0.2	0.2	0.5	0.5	1.0	1.0	0.3	1.0	0.5	0.3	0.1	0.340539	0.951321
										0.3	0.34442	0.845375
										0.6	0.350381	0.693057

**Table 3:** Comparison of Skin-friction with Khan et al. [40].

$W_{1}$	$M_{1}$	Present	Khan et al. [40]
0.1	0.1	2.05600	2.05608
0.2		2.01100	2.01101
0.3		1.96121	1.96124
0.1	0.2	2.14588	2.14587
	0.3	2.23184	2.23184

**Table 4:** Comparison of Nusselt number with Khan et al. [40].

$N_{b}$	Pr	Present	Khan et al. [40]
0.1	1.0	0.4700	0.4701
0.2		0.4411	0.4410
0.3		0.4134	0.4135
0.1	1.1	0.4966	0.4965
	1.2	0.5217	0.5216

## Abbreviations

The following abbreviations are used in this manuscript:

MHD	Magnetohydrodynamics
RK−45	Runge-Kutta 45 Method
ODE	Ordinary Differential Equation
PDE	Partial Differential Equation
u,v	Horizontal and vertical velocity components/m·s${}^{-1}$
x,y	Cartesian coordinates/m
$u_{w}=bx^{n}$	Stretching velocity/m·s${}^{-1}$
μ	Dynamic viscosity/Pa·s
*b*	Stretching rate/s${}^{-1}$
ν	Kinematic viscosity/m${}^{2}\cdot$s${}^{-1}$
$B_{0}$	Applied magnetic field intensity/A·m${}^{-1}$
*k*	Thermal conductivity/W·m${}^{-1}\cdot$K${}^{-1}$
$\rho_{fl}$	Density/kg·m${}^{-3}$
$D_{B}$	Brownian diffusion
$D_{T}$	Thermophoresis
$T,T_{\infty },T_{w},T_{fl}$	Temperature distributions at various locations/K
$C,C_{\infty },C_{w},C_{fl}$	Concentration distributions at various locations/kg·m${}^{-3}$
σ	Electric conductivity of the fluid/(Ω m)${}^{-1}$
${\left(\rho c\right)}_{p}$	Nanoparticles’ productive heat capacity/J·m${}^{-3}\cdot$k${}^{-1}$
${\left(\rho c\right)}_{fl}$	Fluid’s productive heat capacity/J·m${}^{-3}\cdot$k${}^{-1}$
*M*	Magnetic parameter
$C_{b}$	Drag force coefficient
*K*	Permeability of the porous medium
τ	Ratio of heat capacity of fluid and nanoparticles
$K_{r}$	Binary chemical reaction
*E*	Activation energy
**Dimensionless Parameters:**	
Sc	Schmidt number
Pr	Prandtl number
$N_{t}$	Thermophoresis
$N_{b}$	Brownian diffusion
$Nu_{x}$	Nusselt factor (Heat flux parameter)
$Sh_{x}$	Sherwood factor (Mass flux parameter)
$W_{1}$	Weissenberg number
$N_{G}$	Entropy generation rate
$\beta_{1}$	Temperature difference parameter
$\beta_{2}$	Concentration difference parameter
$Br_{1}$	Brinkman number
$L_{1}$	Diffusion parameter
$F_{r}$	Inertial force parameter
$K_{R}$	Binary chemical reaction parameter
$E_{1}$	Activation energy parameter
$E_{G}$	Entropy generation
λ	Porosity factor
η	Variable
$f^{\prime }$	Velocity
θ	Temperature
ϕ	Concentration
